# Performance evaluation of an algorithm for fast optimization of beam weights in anatomy-based intensity modulated radiotherapy

**DOI:** 10.4103/0971-6203.62203

**Published:** 2010

**Authors:** Vaitheeswaran Ranganathan, V. K. Sathiya Narayanan, Janhavi. R. Bhangle, Kamlesh K. Gupta, Sumit Basu, Vikram Maiya, Jolly Joseph, Amit Nirhali

**Affiliations:** Healthcare Sector, Siemens Ltd, 403A, Senapati Bapat Road, Pune- 411 016, India; 1 Department of Radiation Oncology, Ruby Hall Clinic, 40-Sasoon Road, Pune-411 001, India

**Keywords:** Anatomy-based MLC fields, aperture-based IMRT, Gaussian Elimination method, IMRT

## Abstract

This study aims to evaluate the performance of a new algorithm for optimization of beam weights in anatomy-based intensity modulated radiotherapy (IMRT). The algorithm uses a numerical technique called Gaussian-Elimination that derives the optimum beam weights in an exact or non-iterative way. The distinct feature of the algorithm is that it takes only fraction of a second to optimize the beam weights, irrespective of the complexity of the given case. The algorithm has been implemented using MATLAB with a Graphical User Interface (GUI) option for convenient specification of dose constraints and penalties to different structures. We have tested the numerical and clinical capabilities of the proposed algorithm in several patient cases in comparison with KonRad^®^ inverse planning system. The comparative analysis shows that the algorithm can generate anatomy-based IMRT plans with about 50% reduction in number of MUs and 60% reduction in number of apertures, while producing dose distribution comparable to that of beamlet-based IMRT plans. Hence, it is clearly evident from the study that the proposed algorithm can be effectively used for clinical applications.

## Introduction

In inverse planning for IMRT, the conventional practice is to define the constraints for tumor and normal tissues based on which the optimal fluence is calculated.[1,2] The optimized fluence is delivered using an ordered MLC shape-sequence, made by a “leaf sequencer program”.[3–5] This method is known as fluence-based inverse planning or Beamlet-based Inverse Planning (BBIP). The main limitation of this method is that the optimization step does not take into account the delivery constraints imposed by MLC. Therefore the delivery fluence maps may differ from the optimized fluence maps due to physical and mechanical constraints of the MLC. This discrepancy degrades the treatment delivery efficiency and also increases the treatment verification load. It may also produce a leaf sequence that generally requires a large number of apertures and monitor units (MUs). Increase in number of MUs increases the whole body dose due to leakage radiation. Various investigators[6–9] and recently Hal[10] have expressed concern over the the greater probability of cancer induction due to increase in the whole body scatter with IMRT.

Several techniques have been proposed to simplify IMRT plans. An alternative approach to BBIP is Aperture-based Inverse planning (ABIP), where only required numbers of deliverable apertures are generated and their weights get optimized. Also, plans with pre-defined anatomy-based MLC fields (anatomy-based ABIP), as proposed by many authors,[11–15] could be considered to reduce both the treatment complexity and verification burden. The optimization of the beam weights in anatomy-based ABIP was addressed by many investigators by using some form of Monte Carlo technique[16–18] simultaneous iterative back projection method[19–21] or other algebraic techniques.[22,23] The efficiency of such approaches or algorithms depends upon the ability to match the BBIP dose distributions along with significant reduction in no. of beam segments and MUs.

In this communication, we present an algorithm to optimize beam weights in anatomy-based IMRT. The distinct feature of the algorithm is that it takes only fraction of a second for optimizing the beam weights, irrespective of the complexity of the given case. The algorithm uses a numerical technique called Gaussian-Elimination that derives the optimum beam weights in an exact or non-iterative way. The algorithm has been implemented using MATLAB with a Graphical User Interface (GUI) option for convenient specification of dose constraints and penalties to different structures. We initially tested the performance of the algorithm in different patient cases in terms of convergence, consistency and optimization speed. We also tested the clinical capabilities of the proposed algorithm in several patient cases. To date, we have treated three patients planned using the algorithm in our clinic. In this article, we limited the clinical performance analysis to two patient cases, performed in comparison with beamlet-based IMRT plans generated using KonRad^®^ inverse planning system.

## Materials and Methods

### Design of anatomy-based MLC shapes

We use a simple anatomy-based segmentation method[11–15] to manually generate the anatomy-based MLC fields. In this method, the first field is adjusted to the projection of the target with an appropriate margin using the standard beam's-eye-view (BEV) display, blocking the organs-at-risk (OAR) present within the BEV of the target results in the subsequent fields per gantry/couch angle combination, depending on the anatomy. In this approach, the intensity of each aperture is allowed to vary continuously. In combination, these apertures produce complex intensity maps.[24]

### Optimization description

To optimize beam weights in IMRT, the objective is to minimize the difference between the prescribed and calculated dose distributions.[25] The optimization is based on the following quadratic dose-based cost function.

(1) $$\text{F}\left(\text{d}\left(\text{x}\right)\right)=\sum_{\text{i}=1}^{\text{N}}{\text{p}}_{\text{i}}{\left({\text{D}}_{\text{i}}\left(\text{x}\right)-{\text{PD}}_{\text{i}}\right)}^{2}$$

Here F(**d**(**x**)) is the cost function, where **d**(**x**) is a dose distribution among the voxels of the patient model and x is the vector of parameters to be selected (beam weights) and N is the no. of voxels. D_i_ = D_i_(x) and ^p^D_i_ are the calculated and prescribed maximum dose in voxel i, respectively. Here the array P_i_ contains the penalties for different structures for violation of dose limits. For instance, a voxel in the target is penalized if it is overdosed (>D_max_) as well as underdosed (<D_min_). Likewise, a voxel in the normal structure get penalized if it receives a dose more than its *D*_max_.

In order to minimize the cost function, a set of optimum beam weights is to be derived. In our approach, instead of attempting to solve the problem in an iterative manner, an exact analytical model is created that describes the whole optimization problem with a set of linear equations solving which will minimize the cost function for the given dose constraints and penalties. A set of simultaneous linear equations that describes the optimization problem is shown in equation 2:

(2) $$\begin{array}{l} {\text{D}}_{11}{\text{W}}_{1}+{\text{D}}_{12}{\text{W}}_{2}+\ldots \ldots \ldots \ldots \ldots \ldots {\text{D}}_{1\text{n}}{\text{W}}_{\text{n}}={\text{d}}_{1}\\ {\text{D}}_{21}{\text{W}}_{1}+{\text{D}}_{22}{\text{W}}_{2}+\ldots \ldots \ldots \ldots \ldots \ldots {\text{D}}_{2\text{n}}{\text{W}}_{\text{n}}={\text{d}}_{2}\\ {\text{D}}_{\text{m1}}{\text{W}}_{1}+{\text{D}}_{\text{m2}}{\text{W}}_{2}+\ldots \ldots \ldots \ldots \ldots \ldots {\text{D}}_{\text{mn}}{\text{W}}_{\text{n}}={\text{d}}_{\text{m}} \end{array}$$

Where the general term D_mn_ denotes the dose to an m^th^ voxel from a n^th^ aperture-segment before optimization and W_1_, W_2_…W_n_ are the optimized aperture weights that will produce a prescribed dose of d_m_ at the m^th^ voxel.

In order to solve Equation 2, a Gaussian-Elimination code is written along with additional modules to smoothening of the beam weights. In linear algebra, Gaussian elimination is a simple yet powerful algorithm that can be used to determine the exact solutions of a system of linear equations. Gaussian Elimination derives the solution through a series of parameter elimination and back-substitution processes that derives the result without taking any iteration.[26–29] Our beam weight optimization scheme can be expressed using Equation 3.

(3) $$\text{W}\left(\text{z}\right))=\varphi \left({\text{D}}_{\text{o}}\left(\text{x}\right),{\text{ D}}_{\text{p}}\left(\text{y}\right)\right)$$

where **W** is a 1D array of optimum aperture weights, **D**_0_ is a 2D array of dose values at each voxel due to each aperture before optimization, and **D_p_** is a 1D array of dose constraints to each voxel.

### Dose calculation

Our algorithm has been implemented in MATLAB. Patient contours are first generated in the CMS-XiO^®^ (4.3.1) treatment planning system. In this planning system the dose is calculated using a fast convolution superposition algorithm.[30] Also, the user defined anatomy fields are generated and doses are estimated by the planning system initially for a set of equal weightings of the fields. Sample points are then manually distributed in each structure based on the anatomy. Then the dose values obtained at those user defined sample points are loaded into MATLAB for optimization. The main advantage of this approach is that there is no need for separate dose calculation software for optimization. Because, once the optimum beam weights are calculated for a given set of dose constraints and penalties, the corresponding dose values at each sample point can be derived just by reversing the operation of the algorithm. Once the doses at each sample point are obtained, a subroutine incorporated with the algorithm immediately calculates the current cost function value. After the final optimization, the resulting beam weights are again exported to CMS-XiO planning system and the dose is recalculated in order to confirm that the dose values derived by our algorithm are accurate. The DVHs, dose distributions and MU calculations presented in the paper were calculated using CMS XiO and KonRad planning systems.

### Graphical user interface

As part of the work, a Graphical user Interface (GUI) was developed. The GUI was written in MATAB with DICOM compatibility for enabling communication between the planning system and the algorithm. The GUI contains a series of push buttons, checkboxes and pop-up menus to implement the following functions: (1) import: importing dose values obtained at sample points; (2) Optimization parameters: inputting dose constrains and penalties for different structures; (3) Optimization process: performing optimization and re-optimization; and (4) Output: display of the final beam weights and cost values.

### Numerical performance analysis

By far, applying of Gaussian Elimination in optimization of IMRT has not been attempted by any researchers, because the performance of such non-iterative algorithms may degrade for large scale optimizations problems (E.g. 10^4^ - 10^6^ simultaneous equations). However, the speed and accuracy of Gaussian Elimination method for relatively small (E.g. 10^1^ - 10^2^ equations) and medium (E.g. 10^2^ - 10^4^ equations) scale optimization problems can be better than any iterative method. In order to verify the actual numerical capability of the algorithm in the context of beam weight optimization, an analysis was initially performed for a variety of patient cases, in which we evaluated the characteristics of the algorithm in terms of convergence, consistency and speed. To perform the analysis, different data sets (A, B, C, D, E, F, G and H) are generated that belong to different patient cases (H&N 1, H&N 2, Brain 1, Brain 2, Brain 3, Abdomen 1, Abdomen 2 and Abdomen 3 respectively). Each data set is represented using a cost function, whose basic structure resembles the one shown in equation 1. The data sets comprise the user specified treatment parameters (no. of gantry angles, no. of apertures, etc) and optimization parameters (dose constraints and penalties). The number of gantry angles, number of apertures and their shapes were adapted to the anatomy of the given case as described in earlier Section. Then the dose values obtained at the user defined points were loaded into MATLAB for optimization. A Java-based Data Base Management System (DBMS) was separately used for maintaining the data sets with appropriate connectivity to the optimization algorithm. Then the whole problem was described using a set of linear simultaneous equations (as shown in equation 2). Then we attempted to minimize those cost functions by solving the linear equations (optimizing beam weights) for each data set.

### Clinical performance analysis

We have studied the clinical performance of the proposed algorithm in eight patient cases (2 H&N, 3 Brain and 3 Abdomen) out of which three cases planed using the algorithm have been treated in our clinic. In this communication, we have presented a detailed account on the clinical performance analysis done for two patient cases (H&N and brain) planned using the algorithm. We used a sampling density (ρ) of 1 point/cm^3^ (approximately) for which the cost function has roughly converged in most of the patient cases. For the sake of comparison, beamlet-based IMRT plans were also generated for the same cases presented, using the KonRad inverse planning system, which uses a pencil beam algorithm for dose calculation. We used a grid size of 3mm for the dose calculation in KonRad. Also, we used 3 mm CT slices for the study cases. The gantry, collimator and couch angles are kept same for both plans to make the comparison more realistic. We used an intensity level of 7 in the BBIP plans presented in this study. Moreover, the parameters of the objective function are also kept same for both plans, as the clinical objectives for both plans are obviously the same.

#### Case A

The case is a typical concave H and N lesion of volume 379cc encircling spinal cord and surrounded by parotids on both sides. The difficulty in this case is to cover the target with a prescription dose of 50.4 Gy as homogeneously as possible and at the same time maintain the spinal cord D_max_ Strictly within 45 Gy. The treatment goals for this plan are summarized in Table 1. We developed an anatomy-based ABIP plan with seven 6 MV coplanar beams of angles 0°, 45°, 100°, 150°, 210°, 260°, 315°. The number of apertures/gantry angle was set to be 3 to 5 and hence totally 30 apertures were included in the optimization with appropriate collimator angles.

**Table 1:** Summary of treatment goals for the ABIP and BBIP plans for patient cases A and B presented in the paper

*Case*	*Structure*	*Goals*
Case A	PTV	V_50.4 Gy_ >= 95% and
		V_53 Gy_ <= 55 % and
		V_60 Gy_ <= 0%
	Lt. Parotid	V_20 Gy_ < 40%
	Rt. Parotid	V_20 Gy_ < 40%
	Spinal Cord	V_45 Gy_ < 0%
Case B	PTV	V_50 Gy_ >= 95% and
		V_53 Gy_ <=53 % and
		V_60 Gy_ <= 0%
	Brainstem	V_50 Gy_ < 0 % and
		V_35 Gy_ < 45% and
	Chiasm	V_50 Gy_ < 0 %
	Rt. Optic nerve	V_45 Gy_ < 0%
	Lt. Optic nerve	V_50 Gy_ < 0%
	Rt. Lens	V_10 Gy_ < 0%
	Lt. Lens	V_10 Gy_ < 0%

#### Case B

The case is a temporal brain lesion of volume 273cc surrounded by critical organs such as brainstem, chiasm, and optic nerves. The prescription for the PTV was 50 Gy along with tight constraints to the nearby OARs. The difficulty in this case is because of the close proximity of the target to optic chiasm, left optic nerve and brainstem. Our intension was to give uniform target coverage and reduce D_max_ of chiasm and brainstem to less than 50 Gy. The treatment goals for this plan are summarized in Table 1. We used a non-coplanar beam arrangement with seven 6 MV beams in ABIP and BBIP plans. We set three to five apertures per gantry angle in ABIP plan that leads a total of 28 apertures.

## Results

### Numerical performance analysis

**Figure 1:**
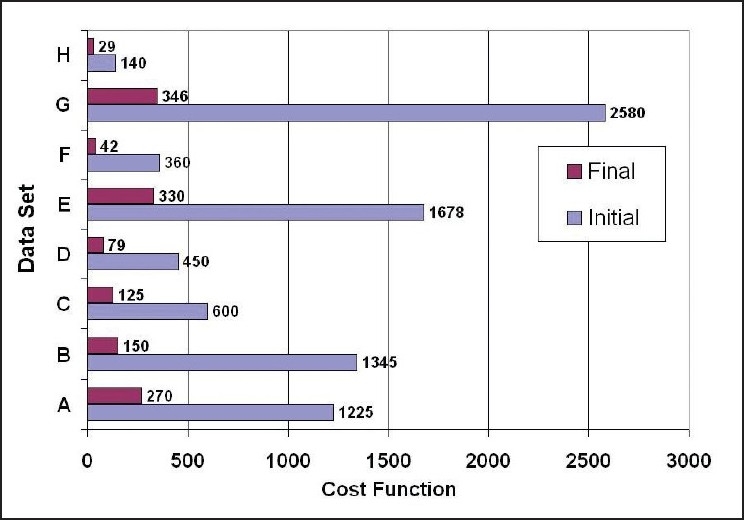
The initial and final cost values obtained after performing a single optimization trial with the algorithm in different data sets

Table 2 shows the result of the numerical performance analysis in detail, performed in different data sets/patient cases. The reduction in the cost function in all data sets is illustrated in Figure 1. The data sets A and E actually represent the Patient Cases A and B taken for the clinical analysis described earlier section. In patient cases A and B, the percent reduction in the cost function is 78 and 80 respectively. Such a reduction in the cost function in both cases resulted in a nearly acceptable dose distribution. It is admitted that the percent reduction in the cost function value does not necessarily mean an equal amount of reduction in the required outcomes. However, in a single criteria optimization, the reduction in the cost function can be used as an approximate indication of the corresponding improvement in the dose distribution.

**Table 2:** Summary of results obtained in the numerical performance analysis

*Data Set*	*Initial value of the Cost Function*	*No. of gantry Angles*	*No. of apertures*	*Sampling Density (points/cc)*	*Number of Equations*	*Final Value of the cost function*	*Percent Reduction*	*Timea taken (Sec)*
A	1225	7	30	1.11	1200	270	78	1.0
B	1345	7	30	1.13	1223	150	89	1.0
C	600	7	32	1.00	700	125	79	0.7
D	450	7	28	0.95	600	79	82	0.6
E	1678	7	28	1.05	1200	330	80	1.3
F	360	5	24	1.15	1250	42	88	0.6
G	2580	9	34	1.20	1100	346	87	1.4
H	140	7	36	0.90	650	29	79	0.4

^a^CPU time taken in a 1.8 GHz Intel Pentium Microprocessor

In our approach, further improvement in dose distribution is derived in successive trials by modifying the penalties of different sub-objectives according to the situation as usually performed in most of the optimization systems. Moreover, we also investigated the consistency of the Gaussian Elimination method by repeating the optimizations for multiple times. Our observation is that the variation in the results is less than 0.3% in almost all the data sets presented. The time taken for each optimization was of fractions of CPU seconds in a 1.8 GHz Pentium microprocessor. Therefore, it is evident from this analysis that the characteristics of the algorithm are numerically acceptable for a broad category of cases in the context of beam weight optimization in anatomy-based IMRT.

### Clinical performance analysis

#### Case A

**Figure 2:**
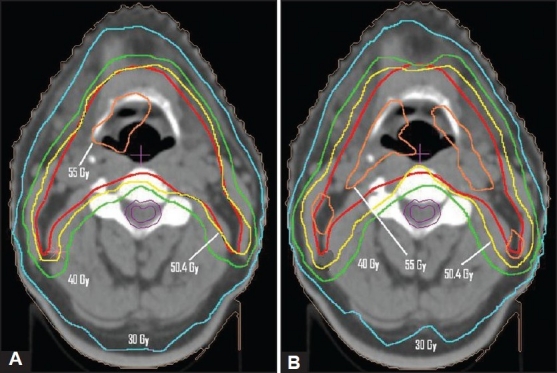
The dose distribution on an axial slice obtained for A) BBIP plan B) ABIP plan obtained in Patient Case A. The solid red line indicates PTV in both plans

**Figure 3:**
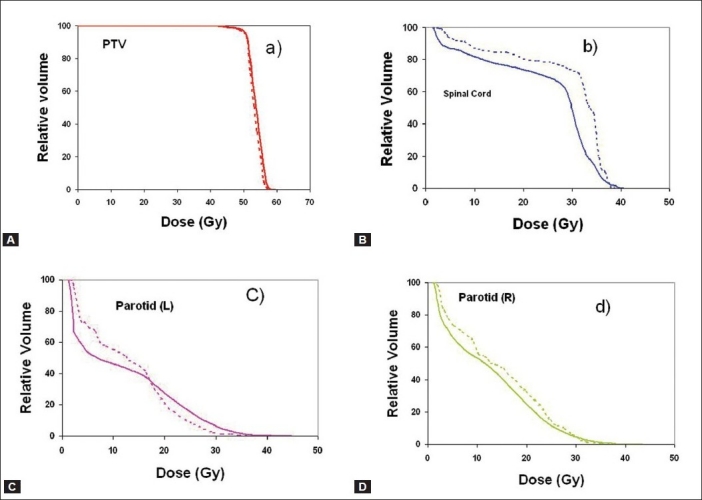
Comparison of DVH plots of ABIP and BBIP plans in patient case A for A) PTV, B) spinal cord and C) parotid (L) and D) parotid (R), where the solid lines denote ABIP plan and dotted lines denote BBIP plan

**Figure 4:**
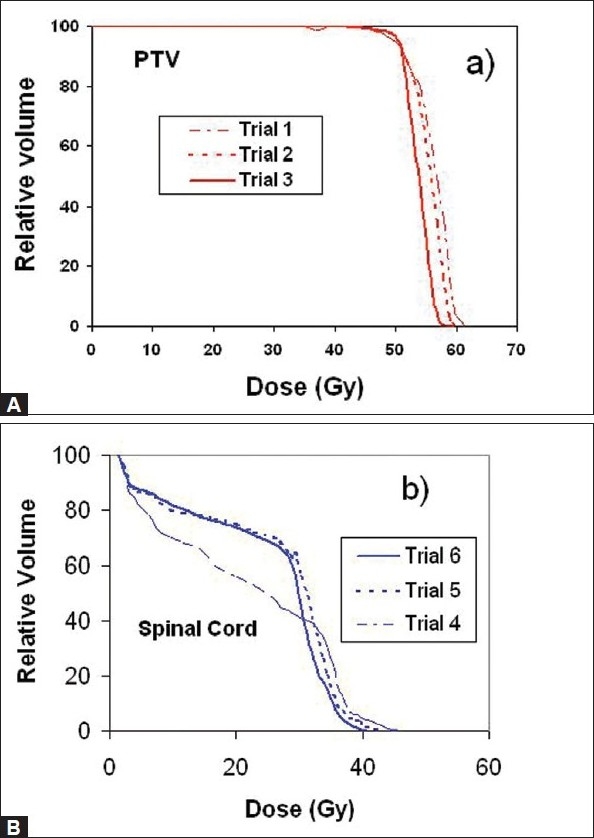
DVHs for A) PTV and B) spinal cord at different optimizations trials for different combinations of penalties in patient case A

Figure 2 presents the dose distribution on a transversal slice for the two plans, namely ABIP and BBIP. Figure 3 shows the dose-volume histograms for the structures of interest, allowing comparison between the two plans. The isodose lines of 50.4 Gy (prescription dose), 40 Gy and 30 Gy are shown in the Figure 2 indicated that the required curvatures in the dose distributions are obtained in ABIP plan as well. Table 3 summarizes the results in terms of dose-volume indices for both plans. The total MU in ABIP and BBIP plans are 360 and 768 respectively. The total beam segments in ABIP and BBIP are 30 and 84 respectively. Figure 4 shows the DVHs obtained for PTV and spinal cord at different optimizations trials. In each trial, we changed the penalties of the structures to improve the results. It took 6 optimization trials with different combination of penalties to get an acceptable DVH and dose distribution in ABIP plan.

**Table 3:** Dose-Volume indices obtained in ABIP and BBIP plans for Patient Cases A and B

*Case*	*Structure*	*Parameter*	*BBIP*	*ABIP*
Case A	PTV	V50.4 Gy (%)	95	95
		V53 Gy (%)	52	54.2
		V58 Gy (%)	0.1	0.2
	Lt. Parotid	Mean (Gy)	20.2	20.8
	Rt. Parotid	Mean (Gy)	19.1	18.6
	Spinal Cord	Max (Gy)	38.3	40.2
Case B	PTV	V50 Gy (%)	96.1	95.4
		V53 Gy (%)	50	50
		V58 Gy (%)	0	0.4
	Brainstem	Mean (Gy)	32.4	31.2
		Max (Gy)	45.7	47.6
	Chiasm	Max (Gy)	45.4	47.2
	Rt. Optic nerve	Max (Gy)	29.8	30.1
	Lt. Optic nerve	Max (Gy)	43.3	45.1
	Rt. Lens	Max (Gy)	3.2	3.4
	Lt. Lens	Max (Gy)	6.4	6.5

#### Case B

**Figure 5:**
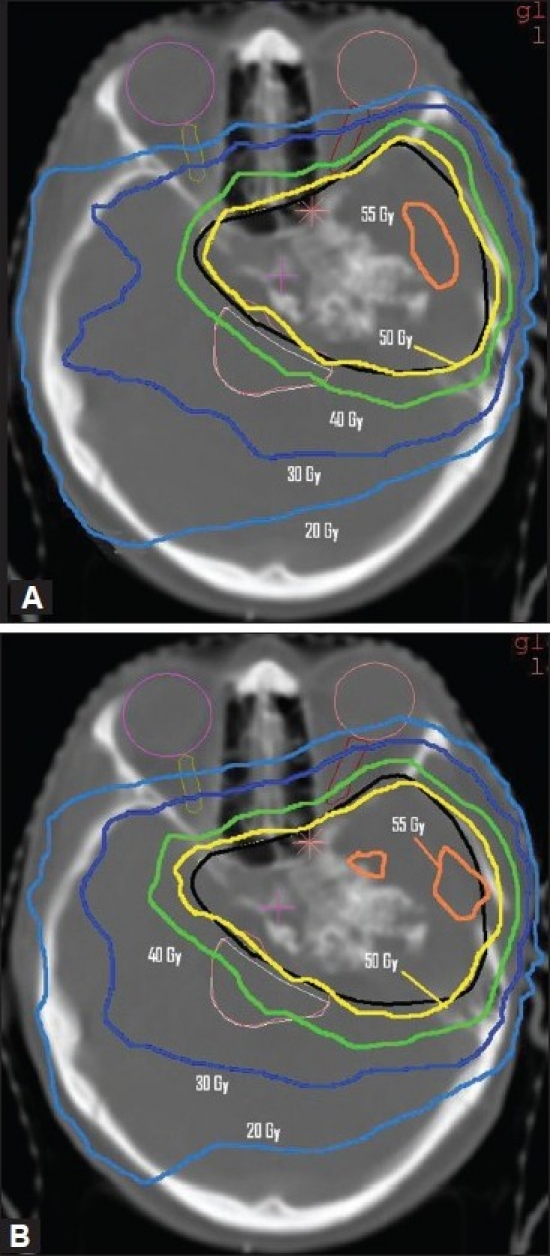
The dose distribution on an axial slice for A) BBIP plan B) ABIP plan obtained in Patient Case B. The solid black line indicates PTV in both plans

**Figure 6:**
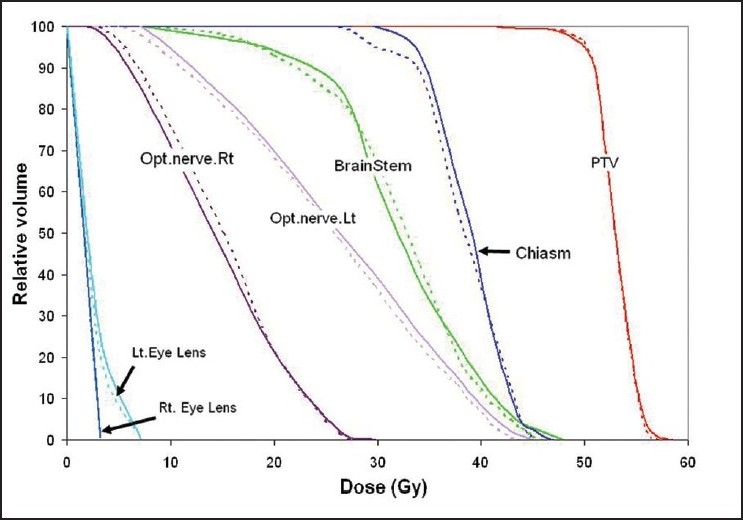
Comparison of DVH plots of ABIP and BBIP plans obtained in Patient Case B, where the solid lines denote ABIP plan and dotted lines denote BBIP plan

**Figure 7:**
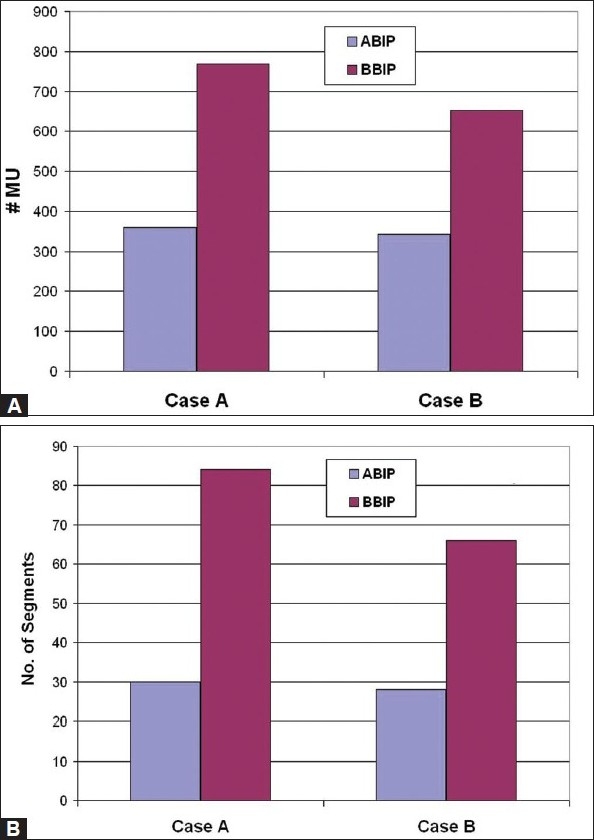
Comparison of A) Number of MUs and B) number of apertures in ABIP plans and BBIP plans obtained for Patient Cases A and B

**Figure 8:**
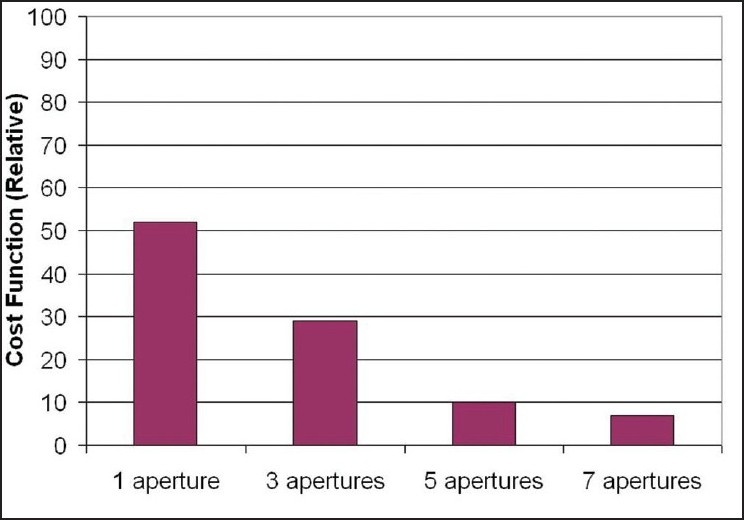
An illustration of the final cost values obtained in Patient Case B with different number of apertures per gantry angle

Figure 5 presents the dose distribution on a transversal slice for the two plans and Figure 6 shows the dose-volume histograms for the structures of interest. The isodose lines of 50 Gy (prescription), 40 Gy, 30 Gy and 20 Gy are shown for both plans in Figure 7 indicate that ABIP plan is comparable to BBIP plan in terms of target coverage, OAR sparing and spillage control. Table 3 summarizes the results in terms of dose-volume indices for both plans. The total MU in ABIP and BBIP is 342 and 652 respectively. The total beam segments in ABIP and BBIP are 28 and 66 respectively. Figure 8 illustrates how the difference in the no. of apertures/gantry angle is reflected in the final cost value. Acceptable dose distribution was obtained only above 3 apertures/gantry angle. Also, it took nine optimization trials in the ABIP plan to get a clinically acceptable dose distribution.

## Discussion

The method described here looks attractive in many aspects. Since the algorithm optimizes beam weights in fractions of a second, the user is given an opportunity to repeat the optimization multiple times with different combinations of penalties by analyzing a plan in multiple clinical, physical and technical viewpoints in a shorter time. The method offers a GUI option for conveniently specifying different dose limits for the tumor and normal tissues as well as assigning individual penalties for reaching the specified objectives. Another advantage in our optimization method is that we did not employ a separate dose calculation algorithm for optimization. We use an existing treatment planning system (CMS XiO) to calculate dose at the user defined points for a set of uniform beam weights and use those dose values as a standard reference. Afterwards, the dose values resulting from each optimization are derived by just performing an inverse Gaussian Elimination operation over those pre-generated reference dose values. This approach reduces a significant amount of time during optimization and allows to directly utilizing the dose calculation accuracy of the existing planning system for the optimization.

It has been clearly demonstrated in the numerical analysis that the numerical performance of the algorithm in terms of convergence, consistency and speed are suitable to perform beam weight optimization in anatomy-based IMRT for a wide range of patient cases. The clinical performance analysis shows that the required concavities are produced in the isodose lines of interest so that it conforms to the tumor volume while sparing OARs effectively. Also it is evident from the study that the ABIP plans generated using the algorithm are comparable to BBIP plans in terms of target coverage, OAR sparing and spillage. However, it can be observed from the clinical results that in some parameters ABIP plans are ranking behind BBIP plans. For instance, in Case A, the PTV D_mean_ is about 4% higher in ABIP plan as compared to BBIP plan and the spinal card D_max_ in ABIP is 5% higher in ABIP plan than BBIP plan. Likewise, in Case B, the D_max_ of chiasm, brainstem and Lt. Optic nerve was about 4% higher in ABIP plan as compared to BBIP plan. However, all the parameters were stringently kept within the desired limits in ABIP plans for both the cases. As suggested in recent investigations,[31,32] it will be necessary to optimize the beam orientations, couch and wedge angles to reach more competitive plans.

The number of MUs and apertures are significantly lesser in the ABIP plans as compared to their BBIP counter plans. For instance, in Case A, the reduction in the number of MUs and segments in ABIP plan was 53% (BBIP:768/ABIP:360) and 64% (BBIP:84/ABIP:30) respectively as compared to BBIP plan. Likewise, in Case B, the reduction in MU and no of segments in ABIP plan was 48% (BBIP:652/ ABIP:342) and 57% (BBIP:66/ABIP:28) respectively as compared to BBIP plan. The observed reduction in the number of MUs and apertures in the ABIP plans can significantly improve the treatment delivery efficiency and reduce the treatment verification burden.

It is accepted that the optimization methodology mentioned in this study is designed only for quickly finding a local optimum instead of locating a global optimum. But, in the cases studied, we could not observe any particular problem in producing desired results by not getting the global optimum in the optimization. This demonstrates that locating a global optimum for the underlying non-convex optimization problem may not be necessarily of any significant importance, at least from a clinical point of view.

## Conclusion

An algorithm for fast optimization of beam weights in anatomy-based IMRT has been proposed. The numerical as well as clinical performance of the algorithm has been investigated in different patient cases. Our results show that one is able to generate anatomy-based IMRT plans using the proposed algorithm that are comparable to BBIP plans in terms of dose distribution and superior to the same in terms of monitor units and number of apertures. It is evident from the study that the proposed algorithm could effectively produce satisfactory plans meeting the clinical objectives, while the verification could remain simple.
